# Forecasting Peaks of Seasonal Influenza Epidemics

**DOI:** 10.1371/currents.outbreaks.bb1e879a23137022ea79a8c508b030bc

**Published:** 2013-06-21

**Authors:** Elaine Nsoesie, Madhav Mararthe, John Brownstein

**Affiliations:** Children’s Hospital Informatics Program, Boston Children’s Hospital, Boston, Massachusetts, USA ; Department of Pediatrics, Harvard Medical School, Boston, Massachusetts, USA ; Network Dynamics and Simulation Science Laboratory, Virginia Bioinformatics Institute, Virginia Tech, Blacksburg, Virginia, USA; Virginia Tech; Assistant Professor of Pediatrics, Harvard Medical School and Harvard-MIT Division of Health Sciences and Technology, Harvard, USA

## Abstract

We present a framework for near real-time forecast of influenza epidemics using a simulation optimization approach. The method combines an individual-based model and a simple root finding optimization method for parameter estimation and forecasting. In this study, retrospective forecasts were generated for seasonal influenza epidemics using web-based estimates of influenza activity from Google Flu Trends for 2004-2005, 2007-2008 and 2012-2013 flu seasons. In some cases, the peak could be forecasted 5-6 weeks ahead. This study adds to existing resources for influenza forecasting and the proposed method can be used in conjunction with other approaches in an ensemble framework.

## Introduction

In a paper published in 1986, Longini et al.^1^ discussed the usefulness of developing approaches to infectious disease forecasting for minimizing the public health impacts of an epidemic. The computational model presented was developed by scientists in the Soviet Union for predicting the spatio-temporal spread of influenza between and within 126 cities and centers in the Soviet Union. The model was based on a system of integro-differential equations with partial derivatives, which were later translated to a set of difference equations for computer analysis. Cities were connected through a transportation matrix with elements representing daily passenger movement between cities. An extension of the model to a global scale was applied to forecasting the worldwide spread of the 1968-1969 Hong Kong influenza A (H3N2) pandemic. Longini et al. 1 concluded that the performance of the model was promising in the forecast of the temporal-geographic spread of influenza over the forecast period, which consisted of 425 days.

Since then several approaches have been proposed for forecasting influenza with varying degree of success. These range from simple compartmental models 2 3 to complex large-scale approaches 4 5. Statistical methods based on the Box-Jenkins approach to time-series analysis and state-space models have also been proposed 6 7 8 9. Several of these approaches aim to forecast different aspects of the influenza epidemic. Predicted measures typically include peak time and height, magnitude and spread. Comparing approaches can be challenging since the gold standard varies and successful prediction is not always clearly defined. However, there have been several achievements in near real-time and retrospective forecasts of peak time, trend and magnitude. These include studies by Towers and Feng 3, which forecasted the 2009 pandemic to peak near the end of October with 95% confidence. Reports from the Center for Disease Control and Prevention (CDC) indicated that the H1N1 peak was observed in the second week of October in the US. Retrospective forecasts by Shaman and Karspeck 10 suggested seasonal influenza peaks could be forecasted in some cases as prompt as 7 weeks before the true peak.

Although, these accomplishments are promising, there are several limitations that impede influenza forecasting. These include limitations inherent in the model assumptions, in addition to challenges incurred in data availability and estimation of disease model parameters during an outbreak. Challenges due to the lack of data for near real-time forecasting are being tackled by the proposal of alternative data sources to augment traditional methods to disease surveillance. One alternative data source is the estimation of influenza activity using search query data. Google Flu Trends (GFT) estimates influenza activity based on a modeling of search queries on terms, which appear to be good indicators of influenza activity. Shaman and Karspeck 10, in addition, to other studies 6 9 have used GFT in influenza forecasting. The method presented by Shaman et al. 10 is more closely related to that presented in this study. However, differences exist in the underlying epidemiology model and the process of parameter estimation. Shaman et al. 10 combine a data assimilation technique (ensemble adjustment Kalman filter) and a simple humidity-forced susceptible-infectious-recovered-susceptible (SIRS) mathematical model. The humidity-driven model is due to findings suggesting that changes in susceptibility and population-contact patterns are not the sole drivers of influenza outbreaks, but also changes in environmental variables such as absolute humidity 11.

In contrast, we present a method, which combines an individual-based model and an optimization approach for influenza forecasting. The individual-based model aims to capture the underlying process of disease transmission based on population contact patterns, which characterizes the dynamics in the observed epidemic time series curve. Individual-based models and other large-scale computational models have been widely used for evaluating control measures for public policy and pandemic planning 12 13. The optimization approach is used to produce possible parameters, which capture the trend observed in the data. Since the process is stochastic, several possible realizations of the epidemic are produced enabling the estimation of confidence bounds for the predicted measures, which are essential for forecasting. For simplicity, we focus on forecasting the peak time. However, preliminary validation of the method’s functionality was applied to forecasting of the peak time, in addition to peak height and magnitude using synthetic, model-generated epidemic time series curves 14. Here, we forecast and update parameters on a weekly basis. Although applied to forecasting influenza-like illness (ILI), the method is not exclusive to influenza but can be applied to other infectious diseases with similar modes of transmission.

## Materials and Methods

**Data - *Google Flu Trends***

ILI data or data from syndromic surveillance systems, which accurately capture influenza activity at a specific population level, can be used in forecasting the epidemic peak. Typically, data from the U.S. Outpatient Influenza-like Illness Surveillance Network (ILINet) provided by the Centers for Disease Control and Prevention (CDC) is considered the gold standard. However, limitations exist in the availability of the data in near real-time. The data is usually subject to retrospective revisions as reports on ILI cases are updated and also publicly released with a time delay. In addition, data is currently not available at the necessary resolution (city and surrounding metropolitan regions) needed for the individual-based model. We therefore opt to use GFT data, which is one of several alternative data sources shown to provide reasonable estimates of influenza activity. The data is provided at a weekly resolution, is openly available in near real-time for several major cities in the US and is usually not retrospectively updated. The process of constructing GFT is formally described in 15. Similar to Shaman et al. 10, we use GFT data for the 2004-2005, and 2007-2008 influenza seasons. We also attempt to forecast the peak of the 2012-2013 influenza epidemic. Peak times for these three epidemics are forecasted for Seattle, Washington.

**Simulation Optimization Approach**

The simulation optimization approach consists of two parts: a stochastic individual-based epidemiology model for simulating influenza-like disease transmission and an optimization procedure for finding optimal parameters, which capture ongoing disease activity. The optimization approach is used to recursively propose new parameter values, which are evaluated based on simulated outcomes from the individual-based model. We separately describe the individual-based model and optimization method.

**Synthetic Networks and Individual-based Networked Model**

The study of infectious disease dynamics has made significant strides due to factors such as improved computational resources, novel surveillance methods, and improved technological devices for rapid tests and diagnostics. Individual-based models requiring large computational resources have benefited from these advances. These methods have been applied to the study of the socio-temporal transmission and the evaluation of measures for controlling the propagation of infectious disease outbreaks in large populations 12. The overall approach comprises of two parts: (i) synthesizing a social contact network that captures detailed and time varying interactions between individuals comprising the urban region under consideration and (ii) a high performance computing oriented dynamical model that simulates the spatial disease propagation and efficacy of interventions. The process of synthesizing a social contact network for an urban region is based on our earlier work and can be found in^16^ ^20^ ^27^. The individual-based model used in simulating the spread of disease in this paper was first described in 16. Since, these models are not a novel contribution of this paper and has been thoroughly described in several other publications, we therefore summarize both the components briefly; see 17 18 19 27 for additional details.

Synthetic social contact networks for an urban region are constructed using a variety of open source and commercially available data combined with social and behavioral theories. A synthetic social contact network of an urban region is a particular kind of “random network” that preserves anonymity and privacy of individuals and yet is statistically similar to a realistic social contact network. It is important to note that such networks cannot be obtained by simple measurements alone.

First, a large time-scale is associated with land use and demographic distribution as a characterization of travelers including their spatial distribution. In this phase, a synthetic population is created. The synthetic population is a set of synthetic people, each associated with demographic variables drawn from demographical information available in the US census. Joint demographic distributions can be reconstructed from the marginal distributions available in typical census data using the *iterative proportional fitting* technique. Each synthetic individual is placed in a household with other synthetic people and each household is located geographically in such a way that a census of the synthetic population yields results that are statistically indistinguishable from the original census data if they are both aggregated to the block group level.

Next, a set of activity templates for households are determined, based on several thousand responses to an activity or time-use survey. These activity templates include what sort of activities each household member performs and what time of day they are performed. Each synthetic household is then matched with one of the survey households, using a decision tree based on demographics such as the number of workers in the household, number of children of various ages, etc. The synthetic household is assigned the activity template of its matching survey household. For each household and each activity performed by this household, a preliminary assignment of a location is made based on observed land-use patterns, tax data, etc. This guess must be calibrated against observed travel-time distributions. Combining these steps, one obtains a synthetic representation of individuals in an urban region carrying out daily activity patterns at realistic spatial locations.

This information can be abstractly represented by a (vertex and edge) labeled bipartite graph G_PL_, where *P* is the set of people and *L *is the set of locations. If a person \begin{equation*}\small{p \in P}\end{equation*}visits a location \begin{equation*}\small{l \in L}\end{equation*} ** **, there is an edge (*p, l, label*) \begin{equation*}\small{\in}\end{equation*} E(G_PL_) between them, where *label* is a record of the type of activity of the visit and its start and end points. Each vertex (person or location) can also have labels. A person’s labels correspond to his/her demographic attributes such as age, income, etc. The labels attached to locations specify the location's attributes such as its *x *and *y* coordinates, the type of activity performed, maximum capacity, etc. Note that there can be multiple edges between a person and a location recording different visits. We use the term *people-location-visitation graph* to refer to the above bipartite graph, wherein multiple edges are discarded and time labels are omitted. Several projections of the bipartite graphs are possible. The *people-people-spatial-proximity* graph has as its vertices individual people in an urban region and an edge between two people denotes that the individuals came within certain spatial proximity during the course of the day. The modeling approaches used in constructing this model can be found in several publications. See^ 20^ , and 21 , and 22 for information on urban population mobility models. See 13 23 24 25, and 26, for information on disease transmission models and the natural history of the disease. For further information on contact networks, see 13 27, and 28.

As stated, the individual-based model consists of the dynamic social contact network and an individualized disease model. The within host disease model is based on a **S**usceptible, **E**xposed, **I**nfectious, **R**ecovered (SEIR) representation. Each infected agent progresses through the different transmission states based on defined incubating and infectiousness time periods. The incubation and infectious periods are described using discrete probability distributions. Transition between states can be affected by the attributes of the individuals (such as age, and health status) and the type of contact (casual, or intimate). The probability of transmission between susceptible (u) and infectious (v) individuals is given by:

p(w(u,v))=1 −(1 −r)^w(u,v)^

Here w(u, v) represents the contact duration and r is the disease transmission rate, which is defined per sec/contact time. We have one such model per individual. These individualized models are connected based on the people-people proximity graph described above. The networked model is too complex to study analytically. Over the last several years, faster simulations have been progressively developed to study the dynamics of disease spread. Here we use a modeling environment called Epifast. Epifast can simulate disease dynamics over a large social contact network in a matter of minutes. It also has the ability to realistically represent natural intervention strategies. To simulate epidemics, the population, disease characteristics and initial conditions, such as the number of initially infected individuals are selected. Published studies have validated different components of the model. Examples illustrating structural validity include 17 27 and 13.

***Parameters***

The SEIR model requires three disease parameters: incubation period, infectious period and transmissibility. All other parameters are assumed fixed. Both the infectious and incubation periods are defined as discrete probability distributions. Individuals in the synthetic population have a probability of 0.3, 0.5 and 0.2 of experiencing an incubation period of 1, 2, or 3 day(s) respectively. Similarly, individuals can also have an infectious period of 3, 4, 5, or 6 days with probabilities 0.3, 0.4, 0.2 and 0.1 respectively. The mean incubation and infectious durations are therefore individually 2 and 4 days. These and similar parameters have been used in several studies on seasonal influenza dynamics 13 29.

In the individual-based model, the transmissibility parameter is defined as the probability of transmission per unit of contact time given contact between an infectious and susceptible individual. In this study, we limit parameter estimation to the disease transmissibility, although the overall approach is designed to forecast the epidemic curve based on estimation of these three parameters. Since we are solely forecasting seasonal epidemics, we assume that the incubation and infectious periods are consistent. We also assume that in addition to changes in environmental conditions and contact patterns, variability in transmission influences peak time. Studies have indicated that influenza epidemics with higher transmissibility would likely result in higher morbidity, higher peak height and earlier peaks 30 31.

Estimation of the transmissibility of an infectious disease is an important bio-surveillance issue especially for outbreaks such as influenza, which have a mean serial interval shorter than that of most infectious diseases 32. In addition to the serial interval, other measures such as the basic and effective reproduction numbers are typically used in estimating disease transmissibility. We estimate the model transmissibility parameter using a simulation optimization approach. The proposed transmissibility is used in epidemic simulation and forecast of peak time.

***Simulation Initialization***

The individual-based model simulates disease spread on a daily time scale, while GFT data is presented at a weekly resolution. Assuming that infections at time *t* are a result of infections at previous time steps *1…t-1*, we need to simulate approximately the same number of ILIs during the first week as observed in week one of the GFT time series curve. However, we seed the individual-based model with infected persons on day one. Taking into consideration that synthetic individuals in the network can have different infectious and incubation periods and infectiousness can last up to 6 days, we search for a possible initial seeding scenario. We randomly infect different number of individuals on day one and compare the resulting weekly ILI to that observed in the GFT data. This process is evaluated using data from the 2004-2005 influenza season. We decide to seed the simulations with an initially infected count of approximately one-fifth of the GFT counts observed in week one. This results in simulated ILI count on week one that is within 5% of that observed in the first week of the GFT data. In addition, to introduce prior immunity into the network, we randomly vaccinate 20% of the synthetic population.

**Parameter Search**

***Optimization Approach***

Several algorithms can be used in the parameter search problem. Here we apply a classical stochastic root finding optimization approach proposed by Robbins and Monro 33 with additional constraints. We illustrate that under certain assumptions the proposed simulation optimization technique can be used in conjunction with the individual-based model to forecast the peak of an ongoing epidemic by minimizing the difference between cumulative infections for the ongoing epidemic and simulated instances for the same time period.

The algorithm can be explained as follows. Let θ represent the transmissibility where

M(θ) = α_t_

Here, M(θ) represents the current cumulative ILI counts as a function of the disease transmissibility θ and α_t_ is the total ILI observed from week *1 . . . t*. Estimated values of θ are expected to vary initially, but converge towards a specific value as the epidemic nears its peak. The definition of week *1 . . . t* is not fixed. Here, we define week one as the first week in October since we construct the epidemic curve using data starting from that week. The algorithm also assumes that M is a monotonic function of θ, which is reasonable since increasing the disease transmissibility also increases the total ILI for a given contact network 30.

The iterative step of the Robbins-Monro algorithm is given by

\begin{equation*}\small{x_{n+1} = x_{n} + a_{n}(\alpha - \tilde{M}(x_{n})) } \end{equation*}

where, \begin{equation*}\small {\tilde{M}(x_{n}) }\end{equation*} is the estimate of total ILI for the transmissibility rate x_n_ and is obtained by simulation. {a_n_} is an appropriately chosen sequence of positive real numbers that satisfy the following conditions.

\begin{equation*}\small{\sum_{n=1}^{\infty}{a_{n} } = \infty}\end{equation*} and \begin{equation*}\small{ \sum_{n=1}^{\infty}{a_{n}^{2}  < \infty}}\end{equation*}

The algorithm terminates when the iterations (set at 5000) are depleted or the percent error is less than the tolerance, which is set at 0.05%. The percent error at time t is defined as:

\begin{equation*}\small{ \rho = (M(\theta) - \tilde{M}(x_{n}))/M(\theta) }\end{equation*}

Here, M(θ) represents cumulative ILI counts for the ongoing epidemic and (x_n_) are the cumulative simulated ILI counts. We also considered using the doubling time, Pearson and Spearman correlation coefficients, but these as well had limitations, and appeared to be affected by slight deviations in the trend of the data.

As stated, the algorithm stops when the number of maximum iterations is reached or the percent error is less than the tolerance. If the number of iterations is depleted before convergence, we randomly select a new initial value in the current path and restart the optimization process. The transmissibility at convergence is used to initialize the forecasting process for the next week. Since the analysis is retrospective, each forecast is assumed to be made at the end of each week. We start the forecasting process on week 8 representing the last week of November. Starting the forecasting process in November seems reasonable since the typical influenza season runs from November to April in the Northern Hemisphere 34. Note that both the objective function and optimization algorithm can be substituted.

## Results

We present results for the 2007-2008 and 2012-2013 influenza seasons. Forecasts are made from the last week of November starting on 11/25/07 and 11/25/12 respectively. As stated, the first week of October is designated as week one. We also considered starting the forecasting process as early as October based on data observed from August to October. However, the forecast accuracy did not improve and in some cases degraded, which is probably due to the noise introduced by off-season influenza cases.

***2007-2008 influenza season***

We present weekly forecasts several weeks before and after the peak starting from the end of November for the 2007-2008 influenza season in Figure 1. The epidemic peak is observed during the week starting on 02/17/2008, which is week 20 of the time series GFT curve. Initially, the forecasting process produces a higher transmissibility since weekly ILI counts are much higher than that produced by the individual-based model. Nevertheless, as the epidemic nears the peak, forecasts of the peak improves. Peak forecasts become stable between weeks 14 and 15, which is five to six weeks before the actual peak. 95% sample confidence intervals (CI) and standard deviations around the mean are presented in Figure 2. The 95% confidence bounds are close to the mean suggesting low variance in the forecasting procedure. On week fifteen (mid January), the mean and median peak forecasts both fall on the actual peak week. The 95% CI forecasts the epidemic peak between early to mid February, which agrees with the true peak week of 02/17/2008. The true peak is also captured within peak ranges observed on weeks 13 and 14 (per Figure 1).

**Peak forecast for the 2007-2008 influenza season using GFT for Seattle, WA.**
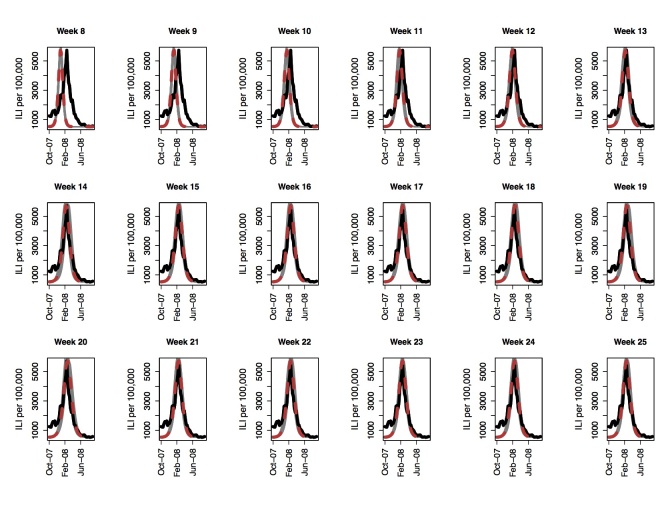
Actual peak is observed on week 20. The black curve is the GFT data, the red line is the mean predicted curve and the grey curves show fifty replicates of the stochastic process.

**Predicted peak by week of forecast for Seattle, WA.**
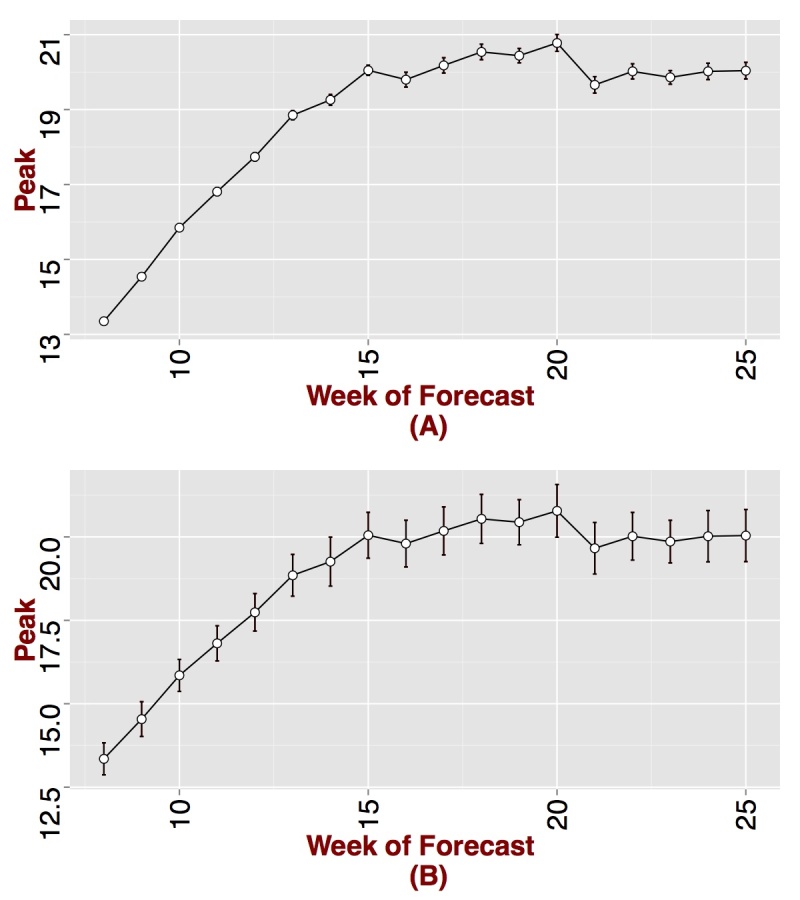
(A) 95% CI around the mean and (B) standard deviations around the mean. The true peak is observed on week 20.

***2012-2013 influenza season***

The 2012-2013 influenza season was more severe than the last five previous seasonal influenza epidemics. The GFT peak was observed during the week starting on 01/13/2013, which is the 15^th^week for the GFT time series curve used in forecasting. Similar to the 2007-2008 epidemic, we start forecasting on week 8. Per Figure 3, peak forecasts improves over time, becoming stable between weeks ten to eleven, which is 4 to 5 weeks from the peak. By week 11, we are 95% confident that the peak would be observed between weeks 14 and 15 (see Figure 4). Similar to observations for 2007-2008 influenza season, the variance around the forecasted mean peak is small. Results observed for 2012-2013 suggests that if GFT data captures the epidemic trend but overestimates the peak, the data could still be used in forecasting the peak.

**Peak forecast for the 2012-2013 influenza season using GFT for Seattle, WA.**
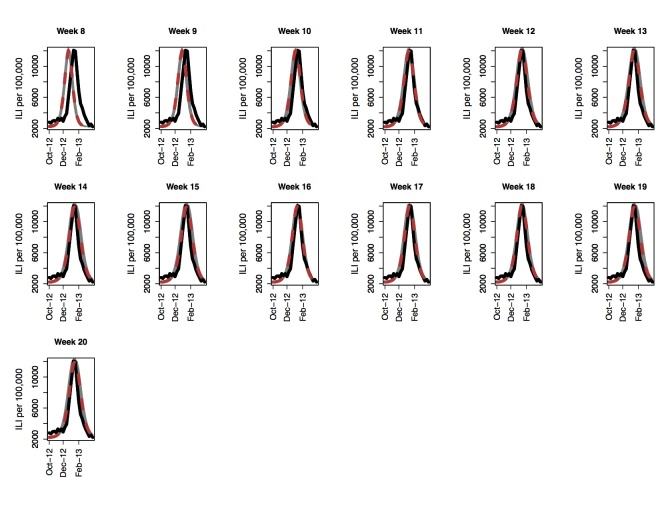
Actual peak is observed on week 15. The black curve is the GFT data, the red line is the mean predicted curve and the grey curves show fifty replicates of the stochastic process.

**Predicted peak by week of forecast for Seattle, WA.**
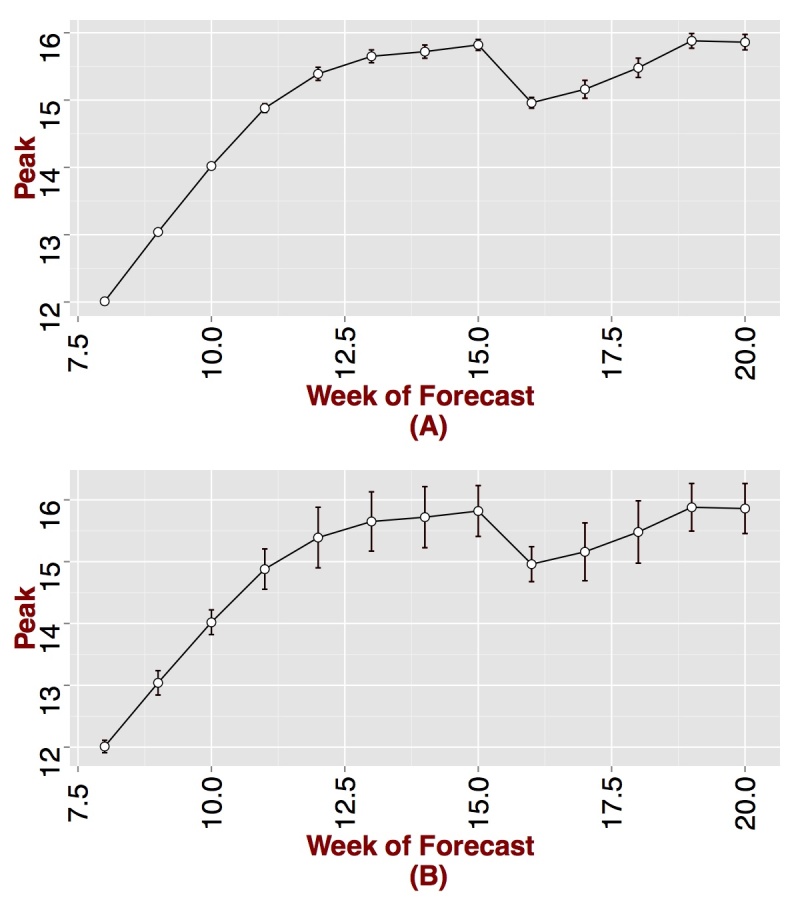
(A) 95% CI around the mean and (B) standard deviations around the mean. The true peak is observed on week 15.

## Discussion

Reliable forecasts of influenza events could influence the allocation of public health resources and control measures. In this initial study, we present a simulation optimization approach for forecasting the peak of seasonal influenza epidemics. The method presented in this study is based on the idea that by using parameters based on the natural history of influenza, epidemics similar to seasonal outbreaks can be produced using the individual-based model. The model disease transmissibility is estimated by recursively proposing new values, simulating epidemics and evaluating the difference between the cumulative illness of the seasonal epidemic and the simulated cases. The transmissibility at convergence is used in forecasting. Different aspects of the model can be replaced; including the optimization algorithm, and the function minimized.

Data from GFT, which estimates weekly ILI counts per 100,000 persons, is used in forecasting. The results are presented for Seattle, Washington for the 2007-2008 and 2012-2013 influenza seasons. Although the overall concept of minimizing the difference between cumulative ILI counts and simulated instances is relatively simple, the observed results are promising. The peak is predicted in one case as early as 5-6 weeks before the actual peak and in another, as early as a month.

As noted in the results, there are deviations in how early the peak can be forecasted by influenza season. These differences could also be observed by region. In this study we used a social contact network developed based on census data for Seattle, while the study by Shaman et al. 10 focused on New York. Shaman et al. 10 suggest that the peak could be forecasted as early as 7 weeks before it is observed. Results in this study agree with that observation though contrary to the study by Shaman et al. 10, no climate variables are included in the simulation optimization approach.

In addition to common challenges to influenza forecasting, there are some limitations introduced by the different components of the simulation optimization approach. First, ILI is typically underreported and can result from a variety of etiologies. Different studies have used different approaches for estimating influenza-attributable symptomatic disease from syndromic data and correcting for bias due to underreporting. However, to our knowledge, there are no standard approaches for dealing with either challenge. In converse, underreporting can be assumed constant over time and introduced into the forecasting approach by scaling the model-generated data. Though, deciding on the appropriate scaling factor can also be difficult.

Second, since the simulation optimization approach does not involve a curve fitting step, the shape of the curve is not accounted for, which could sometimes lead to incorrect forecasts of the peak. Third, the individual-based model does not always capture reality. The lack of information on pharmaceutical and non-pharmaceutical intervention coverage and efficacy, which might influence the shape of the epidemic curve are not readily available during an epidemic. In addition, the generation time for influenza has been estimated to be closer to 3 days 36, while that used in the model is approximately five days. Shortening the length of the incubation period used in the model would shorten the generation time. Lastly, GFT is not always guaranteed to be a reliable estimate of influenza activity. Worry and curiosity induced searches could affect the estimated ILI counts if the model is not consistently retuned. Reports from the 2012-2013 influenza season suggest GFT might have overestimated influenza activity 35. GFT data has also been shown to deviate from patterns of true influenza data.

In this study, GFT is used to illustrate the proposed approach. The results indicate that if the overall trend of the epidemic is accurately captured, GFT could be used for peak forecasts as illustrated, but probably not for forecasting other epidemic measures such as peak height and attack rate. Data from the CDC would be preferred for forecasting influenza, however there are limitations that impede the use of such data presently. One major limitation is the lack of data at the city level. Contact networks for the individual-based model are currently available only at the city level. In order to use CDC data at the regional level, we would need to create regional contact networks. This is an endeavor we are interested in pursuing in future studies.

The approach presented in this study can be made more rigorous by incorporating more information about the influenza strain, and environmental variables such as humidity. However, observations in this study agree with other proposed approaches that influenza forecasting is possible and reliable forecasts can be achieved much earlier than expected.
